# Mitochondrial Dysfunction in Genetic and Non-Genetic Parkinson’s Disease

**DOI:** 10.3390/ijms26094451

**Published:** 2025-05-07

**Authors:** Martina Lucchesi, Letizia Biso, Marco Bonaso, Biancamaria Longoni, Bianca Buchignani, Roberta Battini, Filippo Maria Santorelli, Stefano Doccini, Marco Scarselli

**Affiliations:** 1 Department of Biology, University of Pisa, 56127 Pisa, Italy; martina.lucchesi@biologia.unipi.it; 2 Department of Translational Research and of New Surgical and Medical Technologies, University of Pisa, 56126 Pisa, Italy; l.biso@studenti.unipi.it (L.B.); m.bonaso@studenti.unipi.it (M.B.); biancamaria.longoni@unipi.it (B.L.); bianca.buchignani@fsm.unipi.it (B.B.); marco.scarselli@unipi.it (M.S.); 3 Department of Developmental Neuroscience, IRCCS Stella Maris Foundation, 56128 Pisa, Italy; roberta.battini@fsm.unipi.it; 4 Department of Clinical and Experimental Medicine, University of Pisa, 56126 Pisa, Italy; 5 Molecular Medicine for Neurodegenerative and Neuromuscular Diseases Unit, IRCCS Stella Maris Foundation, 56128 Pisa, Italy; filippo.santorelli@fsm.unipi.it

**Keywords:** Parkinson’s disease, mitochondrial dysfunction, genetic PD, neurotoxins, oxidative stress

## Abstract

Mitochondrial dysfunction is a hallmark of Parkinson’s disease (PD) pathogenesis, contributing to increased oxidative stress and impaired endo-lysosomal-proteasome system efficiency underlying neuronal injury. Genetic studies have identified 19 monogenic mutations—accounting for ~10% of PD cases—that affect mitochondrial function and are associated with early- or late-onset PD. Early-onset forms typically involve genes encoding proteins essential for mitochondrial quality control, including mitophagy and structural maintenance, while late-onset mutations impair mitochondrial dynamics, bioenergetics, and trafficking. Atypical juvenile genetic syndromes also exhibit mitochondrial abnormalities. In idiopathic PD, environmental neurotoxins such as pesticides and MPTP act as mitochondrial inhibitors, disrupting complex I activity and increasing reactive oxygen species. These converging pathways underscore mitochondria as a central node in PD pathology. This review explores the overlapping and distinct mitochondrial mechanisms in genetic and non-genetic PD, emphasizing their role in neuronal vulnerability. Targeting mitochondrial dysfunction finally offers a promising therapeutic avenue to slow or modify disease progression by intervening at a key point of neurodegenerative convergence.

## 1. Introduction

Parkinson’s disease (PD) is a neurodegenerative disorder mostly characterized by motor symptoms, such as resting tremor, bradykinesia and rigidity, and by non-motor features that in some cases can even occur before the onset of the conventional symptoms [1]. It is a complex and multifactorial pathology caused by genetic, environmental and unknown factors; about 85–90% of cases are classified as sporadic (or idiopathic, iPD) with uncertain causes and about 10–15% are familial, mainly characterized by monogenic traits, either dominant or recessive [2].

Over the last 20 years, significant progress has been made in identifying monogenic PD-causing gene mutations with Mendelian inheritance, and at least 23 loci and 19 disease-causing genes have been identified, of which 10 are autosomal dominant and 9 are recessive genes [3]. Some of these cause juvenile or early-onset PD, while others lead to late-onset PD, even if in some cases this subdivision is questionable. In addition, other various genetic risk loci have been found in sporadic PD [4,5]. Importantly, these genetic investigations offer some information on the pathomechanisms of PD that go beyond genetic PD and put forward the understanding of this neurodegenerative disorder in all its aspects.

In relation to the pathogenesis of PD, these genetic loci have pointed to the involvement of specific cellular organelles, such as mitochondria. Mitochondria, as cellular powerhouses, constantly supply energy-producing ATP via the respiratory chain (complexes I–V) and generate reactive oxygen species (ROS) as by-products of this process; moreover, they maintain calcium homeostasis and participate in cell death processes, including apoptosis and necroptosis [6]. Their integrity is conserved by various dynamic processes such as biogenesis, fusion, and fission and quality control mechanisms like mitophagy [7]. As a consequence, mitochondria deficits are associated with ROS overproduction, the release of pro-apoptotic proteins like cytochrome c, and the accumulation of toxic elements, including α-synuclein. This protein is a key element in the pathogenesis of PD, which accumulates in mitochondria, interfering with the respiratory chain function and increasing oxidative stress [8].

The accumulation of aberrant or misfolded proteins can be boosted when dysfunctions of mitochondria, together with aberrancies of the endo-lysosomal-proteasome system, create a vicious cycle of impaired clearance of toxic products, greatly contributing to the development of neurodegenerative pathologies [9,10,11].

Mitochondrial dysfunction has been clearly established as a hallmark of genetic PD, since proteins encoded by the autosomal inherited PD genes—like parkin (*PARK2*), PTEN-induced kinase 1 (*PINK1*; *PARK6*), deglycase *DJ-1* (*PARK7*), high-temperature requirement serine protease A2 (*Omi/HTRA2*; *PARK13*), coiled-coil-helix-coiled-coil-helix domain containing 2 (*CHCHD2*; *PARK22*), and vacuolar protein sorting ortholog 35 (*VPS35*; *PARK17*)—are located in the mitochondria and/or deeply involved in several mitochondrial functions, including the process of mitophagy. Other proteins related to PD-causing genes such as α-synuclein (*SNCA*; *PARK1/PARK4*), leucine-rich repeat kinase 2 (*LRRK2*; *PARK8*), ATPase cation transporting 13A2 (*ATP13A2*; *PARK9*), and F-box protein 7 (*FBXO7*; *PARK15*) are also involved in mitochondria activity or clearance, though they control other relevant cellular functions mostly related to the autophagy process to target dysfunctional cargoes to the lysosomes through the ubiquitin proteasome system (UPS) [12].

Besides these well-known disease-causing genes, other atypical genetic forms of PD (e.g., the one caused by polymerase gamma POLG) generally appear at a very early age and alter several cellular activities, including mitochondrial functionality [13].

The relevance of mitochondrial dysfunction has also been confirmed by non-genetic forms of PD, where environmental toxins such as paraquat, a widely used herbicide, and rotenone, a natural pesticide, are mitochondrial toxins inhibiting complex I activity and increasing ROS production [14]. This mechanism of action resembles that of α-synuclein accumulated within mitochondria, which interferes with complex I function through its N-terminal domain [15].

Importantly, dopaminergic neurons, especially those in the substantia nigra pars compacta, are particularly sensitive to oxidative stress, either caused by genetically determined mitochondrial dysfunction or by toxins, due to their high arborization at their terminals and elevated-energy demand [14]. Enhanced oxidative stress levels eventually lead to neurotoxicity and selective loss of these dopaminergic neurons, which represent a widely recognized hallmark of PD progression [16,17].

In summary, the past and current literature has clearly demonstrated the relevance of mitochondria and lysosomes and their interplay in PD pathophysiology. In this review, we decided to focus on mitochondrial dysfunction determined by juvenile-, early- and late-onset PD-causing gene mutations, as well as the ones involved in juvenile genetic atypical Parkinsonian syndromes. Finally, we explored the mechanisms through which the principal PD-causing environmental toxins affect mitochondrial functions. We invite the readers interested in lysosome involvement in PD to read other reviews [18,19,20].

Based on this premise, we conducted a narrative review, searching in PubMed for relevant keywords, such as “genetic AND (Parkinson* OR PD)”, “mitochondria* AND (Parkinson* OR PD”), “toxic AND (Parkinson* OR PD)”, and by using more specific terms, such as “PINK1 AND (Parkinson* OR PD)”, “rotenone AND (Parkinson* OR PD)”, for each gene and toxic substance of interest. Moreover, we used the snowball search strategy to identify other relevant material in the references of found articles. We conducted the search until the 28 February 2025.

## 2. Juvenile- and Early-Onset Genetic PD and Mitochondria Dysfunction

Juvenile- and early-onset genetic PD represent a subset of pathological cases that usually manifest earlier compared to the typical PD age of onset. The boundaries between these two are often arbitrary. Indeed, juvenile-onset PD usually refers to cases where motor symptoms occur before 21 years of age [21]. However, in some cases no actual distinction is made between them, rather choosing to talk about early-onset (before 45 years of age) and young-onset (before 50 years of age) PD [22,23]. Even in this case, some authors do not distinguish between early-onset and young-onset PD [24]. Besides these disputable distinctions and diversity among the different forms, early-onset patients with PD might more frequently show dystonia and motor complication compared to late-onset genetic PD, slower disease progression, and a delayed occurrence of cognitive decline [25,26].

Therefore, we described a list of genes involved in juvenile- and/or early-onset PD, and how their aberrancies result in mitochondrial dysfunctions (Table 1).

### 2.1. Parkin (PARK2)

**Figure 1:**
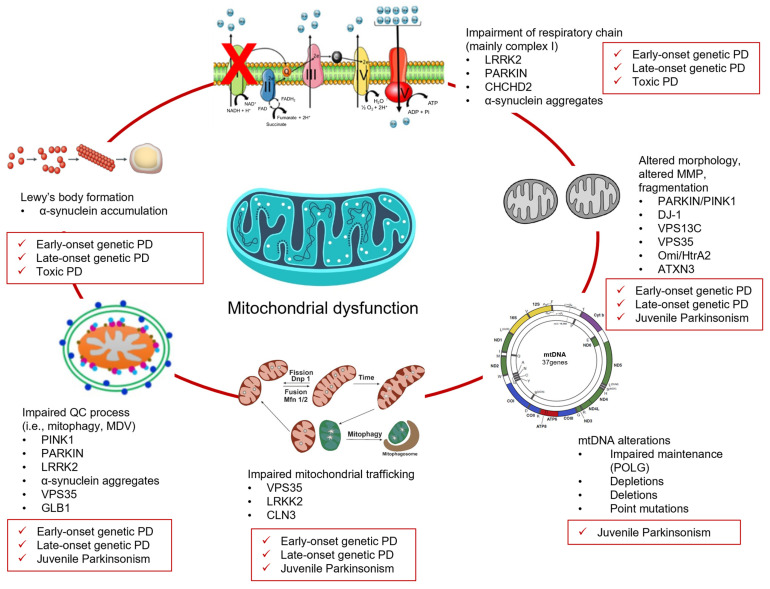
Mechanisms responsible for mitochondrial dysfunction.

Autosomal recessive mutations in parkin (*PARK2*) cause juvenile and early-onset PD [55] with an age onset ranging from 12 to 58 years [56]. In 1997, parkin mutation was linked for the first time to an autosomal recessive juvenile form of PD [57]. Nowadays, more than 130 different parkin mutations have been documented [31], scoring the parkin-related PD as the most prevalent autosomal recessive form of PD [58]. The parkin gene encodes for an E3 ubiquitin ligase, which cooperates with E1 and E2 ubiquitin enzymes for protein degradation through Lys-48-polyubiquitination directing targets to the UPS. Parkin deficiency can impair protein degradation and cause an accumulation of noxious substrates that can be toxic, particularly in the substantia nigra [59]. Moreover, parkin mutants show a decrease in complex I activity, mitochondrial membrane potential (MMP), and cellular ATP levels (Figure 1). This has been evidenced both in fibroblasts derived from patients with PD and parkin mutations [27] and in knockdown zebrafish embryos, characterized by a loss of dopaminergic neurons [29] and by a reduction in complex I and III activity [28]. Compensatory mechanisms can occur to preserve mitochondrial respiratory function, such as complex II-increased activation [36].

Parkin phosphorylation of Ser65 by PTEN-induced putative kinase 1 (*PINK1*) activates its E3 ubiquitin ligase activity, promoting mitophagy [30]. Indeed, Parkin–PINK1 interaction is a determinant for quality control and turnover of mitochondria, including the formation of mitochondrial-derived vesicles (MDVs). A high level of mitophagy mediated by parkin and PINK1 has been observed in mesencephalic dopaminergic neurons [60]. Notably, the involvement of parkin in mitochondrial-derived vesicle (MDV) formation and its influence on antigen presentation suggest immunomodulatory roles that are not typically observed in other PD-related genes.

From a clinical point of view, parkin mutations cause PD characterized by slow progression, dystonia, and typical levodopa response, while non-motor features like olfactory dysfunction and cognitive impairment are less frequent compared to iPD [31].

### 2.2. PINK1 (PARK6)

Autosomal recessively inherited mutations in *PINK1* (PTEN-induced kinase 1), have been recently identified as causative in autosomal recessive early onset parkinsonism (PARK6) with a mean age of onset of 33 years with clinical features similar to iPD [31,61]. Among the 111 *PINK1* mutations identified in PD-affected families, reported variants include point mutations, frameshift mutations, and truncating mutations. PINK1 is a serine-threonine kinase constitutively expressed on the mitochondrial membranes, where it contributes to mitochondria integrity together with the elimination of dysfunctional mitochondria [62]. Under normal conditions, PINK1 is quickly imported into the inner mitochondrial membranes through the translocase (TOM/TIM) complex, where it is processed by the mitochondrial processing peptidase and cleaved by the PARL protease. However, under dysfunctional conditions and oxidative stress, the processing of PINK1 is impaired [30,32] so it accumulates on the outer mitochondrial membrane and facilitates the elimination of damaged mitochondria by phosphorylating different substrates, including parkin and ubiquitin [30,60].

**Figure 2:**
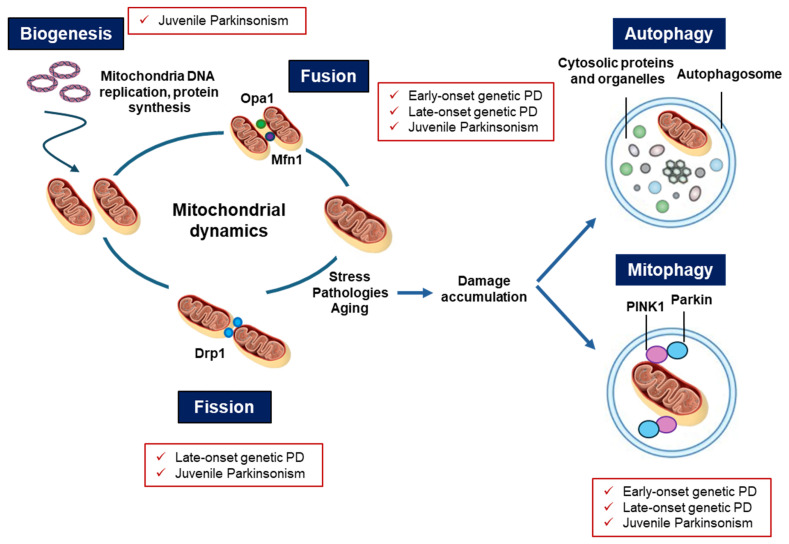
Processes that regulate mitochondrial turnover, such as biogenesis, fusion, fission, mitophagy, and autophagy.

Both PINK1 and parkin seem to serve as instrumental factors for the formation of MDVs, especially under stress conditions, which are fundamental to maintain mitochondrial structure and integrity, and to remove damaged mitochondrial components to lysosomes and peroxisomes for degradation (Figure 2) [63]. Conversely, in another study it was reported that PINK1 and parkin inhibit MDV formation, thus reducing mitochondrial antigen presentation (MitAP) but not affecting basal mitophagy [64]. Thus, both PINK1 and parkin deletions lead to an increase in MitAP and the inflammatory response of immune cells, highlighting potential implications for immune mechanisms in the etiology of PD. This immune-related mechanism appears to be more specifically linked to the PINK1–parkin pathway, as other PD-associated genes more commonly affect mitochondrial bioenergetics or structural maintenance rather than antigen presentation.

Cells expressing parkin and PINK1 mutations either coming from patients with PD or from animal models show decreased activity of oxidative phosphorylation, reduced mitochondrial respiration and mitochondrial morphological abnormalities [27,33,34,35,36]. These aberrancies might depend on the fact that parkin and PINK1 are involved in a common pathway, which influences mitochondrial activity, maintenance and mitophagy [65], especially under oxidative stress. In genetic PD models of drosophila, it has also been observed that PINK1 and parkin mutants have reduced mitochondrial fission, with the consequent enlargement and swelling of mitochondria [66].

Patients with PD and PINK1 mutations have good response to levodopa, as well as dystonia, slow progression, and occasionally psychiatric disorders [67]. Non-motor symptoms such as cognitive dysfunction, sleep, and depression are slightly more frequent compared to Parkin-linked PD, while autonomic symptoms are less common [31,68].

### 2.3. DJ-1 (PARK7)

In 2001, deglycase *DJ-1* (PARK7) mutation was mapped in a family with multiple consanguinity from Netherlands with early-onset parkinsonism [69]; since then, at least 27 variants of *DJ-1* have been registered. The homozygous deletion or point mutation in the human *DJ-1* gene that leads to the replacement of the proline amino acid residue by leucine (L166P) causes an autosomal recessive early-onset form of PD, characterized by slow progression [37]. *DJ-1* is part of the peptidase C56 family, regulated by oxidation on C106, C56, and C46 residues, and it localizes in the cytosol, nucleus, and mitochondria of several human cells [38].

*DJ-1* acts as an oxidative stress sensor and contributes to antioxidant mechanisms, protecting cells from oxidative damage, including dopaminergic neurons which have a high demand for energy produced at the mitochondria level [70]. Once *DJ-1* is activated through oxidation, it translocates into the nucleus where it regulates several transcriptional factors relevant for the redox cellular status, such as the two mitochondrial uncoupling proteins (UCP 4 and UCP5) that reduce MMP and ROS production, the nuclear factor Nrf2, and the pro-apoptotic p53. In addition, it interacts with the mitochondrial protein Bcl-xL, preventing the cytochrome c release and inhibiting apoptosis. Unlike other PD-related genes primarily involved in mitochondrial protein quality control or bioenergetics, *DJ-1* acts predominantly as a redox-sensitive transcriptional regulator, influencing mitochondrial function through cytosolic and nuclear pathways. In vitro and in vivo studies have demonstrated the protective properties of *DJ-1* on dopaminergic neurons against oxidative agents such as hydrogen peroxide, rotenone, paraquat, and 6-OHDA [38,71]. Animal studies on *DJ-1* knockout (KO) models have also highlighted changes in the mitochondrial morphology, particularly in the network connectivity [39]. Consistently, in neuronal cells, the deletion of *DJ-1* altered mitochondrial morphology and it also affected respiratory chain complex integrity [40]. Similar findings were observed in fibroblasts derived from patients with an E64D mutation compared to the controls [72].

Interestingly, oxidized *DJ-1* is reduced in patients with sporadic PD, suggesting possible relevance of this protein for the etiology of certain forms of iPD [70,73].

Patients with PD and with *DJ-1* mutations develop motor symptoms at early stages of the disease and are generally levodopa responders, while later on they can develop amyotrophy, cognitive impairment, and acute behavioral disturbances [37,74].

### 2.4. ATP13A2 (PARK9)

ATP13A2 (PARK9) encodes for a neuronal lysosomal cation-transporting P-type ATPase and loss-of-function mutations of this gene cause diverse neurodegenerative disorders, including an autosomal recessive form of juvenile-onset parkinsonism with dementia (also known as Kufor-Rakeb syndrome) and early-onset PD. Indeed, the age of onset of the disease is typically before 20 years, but it can range from 10 to 33 years [41]. ATP13A2 mutations cause lysosomal and mitochondrial systems impairment, α-synuclein accumulation, decreased mitochondrial clearance, mitochondrial fragmentation, and DNA damage [75]. Recent findings highlighted the involvement of ATP13A2 in counteracting mitochondrial oxidative stress through the mediation of lysosomal polyamine transport, as shown in several models, such as SH-SY5Y cells and in patient-derived fibroblasts [75]. On this line of research, ATP13A2 has been proven to protect against the neurotoxin rotenone, whose toxicity was augmented in ATP13A2-deficient SH-SY5Y cells. In ATP13A2-related fibroblasts derived from patients with PD, there was found a decrease in ATP synthesis, an increase of oxygen consumption, and mitochondrial fragmentation [44].

ATP13A2 is also able to recruit HDAC6 to lysosomes to promote autophagosome–lysosome fusion. On the contrary, in KO mice or in cells lacking ATP13A2, this process is impaired, and the degradation of protein aggregates and damaged mitochondria is reduced, with a consequent accumulation of toxic elements inside the cell, underlining the boundary between lysosomal and mitochondrial activity [76]. This inter-organelle mechanism distinguishes ATP13A2 from other PD-related genes that act primarily within the mitochondria, highlighting its dual role at the interface of lysosomal and mitochondrial quality control.

Lastly, ATP13A2 influences the expression of the mitochondrial transporter TOM20 in the mitochondrial outer membrane [77].

From a clinical point of view, patients with this atypical form of PD are generally responsive to levodopa and they show pyramidal degeneration, spasticity, supranuclear gaze palsy, and dementia [42,43].

### 2.5. PLA2G6 (PARK14)

Although mutations in *PLA2G6* (PARK14) were initially correlated with infantile neuroaxonal dystrophy and neurodegeneration with brain iron accumulation, it was subsequently shown that PLA2G6 gene variants correlate with juvenile- and early-onset PD, usually in the early–mid-20s, with atypical features (dystonia–parkinsonism), and characterized by autosomal recessive inheritance [78]. Nowadays, more than 18 variants are known in unrelated families with different phenotypes [3].

*PLA2G6* encodes for the calcium-independent phospholipase A2β, which is involved in releasing free fatty acids by hydrolyzing the *sn*-2 ester bond in glycerophospholipids [45,79].

The pla2g6 KO model of Drosophila Melanogaster causes mitochondrial lipid peroxidation, mitochondrial dysfunction, reduced ATP synthesis, raised ROS production, reduced MMP, and mitochondrial membrane abnormalities, and these alterations have been confirmed in cultured fibroblasts of patients with PD [46]. Interestingly, lipid peroxidation and the MMP were rescued by treatment with polyunsaturated fatty acids [46]. Similar alterations in mitochondrial structure were found in a pla2g6 KO mouse model [80].

In another study, it was demonstrated that PLA2G6 activates the store-operated calcium signaling (SOCE) and its genetic deficiency causes intracellular calcium depletion and autophagic impairment, particularly in dopaminergic neurons of the substantia nigra, with degenerative consequences [81]. In line with this evidence, PLA2G6 (D331Y) mutation in mice results in the disruption of mitochondria cristae of in dopaminergic neurons of the substantia nigra, with decreased complex I activity, ATP levels, and parkin protein levels, and increased ROS production [82]. Consistently, spinal cord neurons in pla2g6 KO mice show abnormal mitochondria, with degenerated inner membranes, being negative for the inner membrane protein cytochrome c oxidase (CCO) [80,83]. These alterations reflect the distinct role of PLA2G6 in lipid membrane remodeling and calcium homeostasis, setting it apart from other PD-related genes that primarily affect mitochondrial dynamics through ubiquitination or proteolytic pathways.

The clinical features of PD with *PLA2G6* mutations generally are levodopa responsiveness, dystonia, gait impairment, speech difficulties, spasticity, myoclonus, and an association with neuropsychiatric and cognitive disorders [47].

### 2.6. FBXO7 (PARK15)

*FBXO7* (PARK15) mutations are known to cause autosomal recessive juvenile- and early-onset PD with atypical pyramidal symptoms (Parkinsonian-pyramidal syndrome); the median age of onset of the phenotype associated with FBXO7 mutations is 17 years, but it can actually range from 10 to 52 years [84]. In 2008, the autosomal recessive FBXO7 (R378G) mutation was reported in an Iranian family affected from both parkinsonian and pyramidal-associated phenotypes, exhibiting equinovarus deformity since childhood, progressing into pyramidal deficits in the third decade and developing extrapyramidal symptoms later in the most severe cases [48]. So far, three-point mutations in FBXO7 have been observed, while a homozygous truncating mutation and compound heterozygous mutations have been found in other families that present juvenile-onset PD [52].

*FBXO7* gene encodes for an adaptor protein member of the F-box proteins (FBPs) and it is relevant for the Skp1–Cullin–F-box (SCF) ubiquitin E3 ligase activity, involved in the phosphorylation-dependent ubiquitination of targets for proteasomal degradation [49]. Particularly, FBPs determine the specificity of the substrate by bringing it into proximity with the SCF complex for ubiquitination. FBXO7 can also recruit parkin into damaged mitochondria to accelerate the mitophagy process.

FBXO7 deficiency in dopaminergic cells and in patients’ fibroblasts is associated with reduced cellular NAD^+^ and ATP levels, decreased MMP, and impaired activity of complex I. Under these conditions, ROS are increased, and this can trigger the neurodegenerative process, especially in dopaminergic neurons due to their high vulnerability to oxidative stress [50]. Unlike other PD-related genes primarily involved in direct mitochondrial dynamics or oxidative metabolism, FBXO7 modulates mitophagy and cellular energy status indirectly, through its regulatory role in proteasomal targeting and parkin recruitment. Subsequently, ROS induce an excessive activation of poly (ADP-ribose) polymerase (PARP) that normally repairs DNA damage, and this causes the reduction of NAD^+^ and fall in ATP levels [85].

FBXO7 genetic PD is characterized by tremor, rigidity, bradykinesia, pyramidal signs, and varying degrees of levodopa responsiveness, while few patients show cognitive decline [51].

### 2.7. Vacuolar Protein Sorting 13C (VPS13C) (PARK23)

*VPS13C* (PARK23) mutations are associated with autosomal-recessive early-onset PD, rapid progression and Lewy body inclusions. Particularly, the age of onset of symptoms related to *VPS13C* mutations can range from 20s to 40s [53,86]. *VPS13C* truncated mutations were identified for the first time in 3 patients by homozygosity mapping and exome sequencing in families of 1348 unrelated individuals affected by PD [87].

VPS13C belongs to the family of vacuolar protein sorting 13 (VPS13A–D), similar to yeast Vps13p.23, and it is crucial for vesicular transport. Similarly to VPS35, it might be involved in protein trafficking from the mitochondria to peroxisome through MDVs. Intriguingly, *VPS13C* depletion upregulates parkin transcription and exacerbates PINK1/Parkin-dependent mitophagy [88].

In cell models, VPS13C partly localizes to the outer membrane of mitochondria, contributing to mitochondrial maintenance, while its deletion causes lower MMP and mitochondrial fragmentation, with increased respiration rates due to a possible compensatory mechanism. In neuronal cells, such a change in oxidative metabolism could exacerbate the generation of ROS and cause irreversible mitochondrial damage [87,89]. However, recent studies have shown that, different from the other proteins in this family, VPS13C might preferentially be located in the endoplasmic reticulum [90,91]. Moreover, researchers have found that the loss of VPS13C results in alterations in the homeostasis of lysosomes [92]. This multifaceted role across mitochondria, lysosomes, and the endoplasmic reticulum distinguishes VPS13C from other PD-associated genes with more direct roles in mitochondrial dynamics or quality control.

Patients with *VPS13C* mutations have early-onset parkinsonism, usually in early adulthood, cognitive decline, axial symptoms, dysautonomia, and a good response to levodopa treatment in the initial phase but not later as the disease progresses [54,87].

## 3. Late-Onset Genetic PD and Mitochondrial Dysfunction

Late-onset genetic PD typically manifests after the age of 50. Patients suffering from late-onset PD generally have a higher risk for developing cognitive impairment and faster progression compared to early-onset patients with PD [26]. Besides the differences among these late-onset PD-causing gene mutations, the presence of resting tremor suggests a better prognosis [26,93].

Below, we described the monogenic mutations involved in late-onset PD cases, highlighting how they affect mitochondrial homeostasis (Table 2).

### 3.1. SNCA (PARK1/PARK4)

Autosomal dominant *SNCA* (PARK1/PARK4) mutations are widely recognized to cause inherited PD and, moreover, genome-wide association studies have associated sporadic PD cases with some SNCA gene variants. Historically, the first PD-causing *SNCA* point mutation, named p.A53T, was found in an Italian affected by genetic PD, while later on other missense point mutations were discovered (e.g., p.A30P, p.E46K, p.G51D, and p.A53E), usually localized in the N-terminal region of the protein, which is required to bind to neuronal synaptic membrane proteins [94]. In addition to *SNCA* point mutations, duplications and triplications of this gene locus are known to cause inherited PD, with the last condition resulting in the most severe phenotype [95]. The median age of onset of PD caused by *SNCA* alterations is 46 years; however, the specific type of mutation can affect the age of onset. Indeed, triplications cause the earliest onset, with a median age of 39 years (lower quartile of 31 years and upper quartile of 46 years). Duplications result in later onset of symptoms, around 48 years of age (lower quartile of 40 years and upper quartile of 61 years). Point mutations cause an intermediate onset, with reported median ages varying from 43 to 49 years (lower quartile of 42 years and upper quartile of 60 years) [108,116]. In this latter case, the age of onset varies broadly in relation to the specific single nucleotide polymorphism [122].

α-synuclein, a 140-amino-acid polypeptide, is mostly located presynaptically to regulate neurotransmitter release. Importantly, it is the major component of proteinaceous aggregates such as Lewy bodies and Lewy neurites, which are considered pathological hallmarks of PD [123]. α-synuclein is also present in the inner membrane of mitochondria where, through its N-terminal domain, it may affect complex I functions [15]. However, the precise localization of α-synuclein within mitochondria remains debated. While several studies have reported its presence in the inner mitochondrial membrane and interaction with complex I, others have questioned these findings, citing potential artifacts from mitochondrial isolation or overexpression systems [124,125,126]. This ongoing controversy highlights the need for further studies to definitively clarify its intra-mitochondrial distribution and pathogenic relevance. Disruption of this N-terminal domain results in alterations of mitochondria morphology, while the p.A53T mutant mice, in addition to complex I inhibition, have shown altered mitophagy [96]. Furthermore, α-synuclein oligomers bind TOM20, affecting mitochondrial protein import with possible loss of membrane potential and increase in ROS production [127]. In different types of cultured primary neurons, phosphorylated α-synuclein aggregates accumulated in mitochondria cause toxicity, energy deprivation and mitochondrial fragmentation [97]. The interplay between mitochondria dysfunction and α-synuclein is bidirectional and, indeed, metabolic respiratory impairment can also induce α-synuclein mitochondria accumulation. This bidirectional interaction, together with its role in Lewy body formation and synaptic dysfunction, highlights the multifaceted nature of α-synuclein toxicity, which contrasts with the more pathway-specific mechanisms seen in other PD-related genes.

Notably, the presence of oligomeric α-synuclein aggregates compromises SNARE function, decreasing intervesicular space and reducing synaptic vesicles, especially in dopaminergic neurons [128]. Additionally, α-synuclein impairment affects endoplasmic reticulum–Golgi connections, endosomal and autophagosome trafficking, and the autophagosome–lysosome fusion process [128].

Patients carrying SNCA mutations show symptoms similar to iPD including resting tremors, bradykinesia, rigidity, dysphagia, dysarthria, response to levodopa, and a varying degree of cognitive deficits [94,98,129].

### 3.2. LRRK2 (PARK8)

Mutations in leucine-rich repeat kinase 2 (*LRRK2*; PARK8) result in the most common autosomal dominant form of monogenic PD [99,100]. The age of onset can vary between 30 and over 80 years, with a median age of onset of about 58 years. The most frequent mutation is a glycine to serine substitution (G2019S) in LRRK2’s kinase domain, which increases its kinase activity [101]. Additionally, LRRK2 mutations have also been found in sporadic PD, indicating, for some of them, incomplete penetrance [130].

LRRK2 is a multidomain complex whose regions exert different functions, such as GTPase activity, serine/threonine kinase activity and an LRR domain, which allows interactions with several other proteins [131]. For instance, it has been suggested to interact with peroxiredoxin 3 (PRDX3), which is a mitochondrial antioxidant protein [132]. LRRK2 is localized in the mitochondrial outer membrane, suggesting its relevance to mitochondrial functions [133]. In human induced pluripotent stem cell (iPSC)-derived neurons, G2019S mutation has been shown to delay the clearance of dysfunctional mitochondria by stabilizing the microtubule anchor protein Miro which, therefore, remains on damaged mitochondria for longer than in physiological conditions [102,103]. Moreover, LRRK2 G2019S mutant patient-derived fibroblasts showed reductions in cellular ATP levels, with decreased activity of mitochondrial complexes III and IV [104]. LRRK2 is also involved in mitochondrial morphology regulation by interacting with dynamin-related protein 1 (Drp1), and, consistently, Lrrk2 G2019S knock-in mice have shown mitochondrial aberrancies imputable to an arrest in mitochondrial fission [134]. This broad involvement in mitochondrial dynamics, vesicular trafficking, and cytoskeletal regulation underlines the multifunctional nature of LRRK2, setting it apart from PD-related genes with more specialized roles in mitophagy or bioenergetics.

LRRK2 mutations have deleterious effects also on other relevant cellular processes, by influencing vesicular trafficking and lysosome integrity (e.g., enlarged lysosomes), which can lead to cell death, especially in dopaminergic neurons [130]. Moreover, LRRK2 is involved in cytoskeletal maintenance, autophagy immune response [101], and endolysosomal trafficking [135].

LRRK2-related genetic PD is clinically similar to iPD, characterized by bradykinesia, rigidity, resting tremor, gait abnormalities, and postural instability. REM sleep behavioral disorder (RBD) and smell reduction are less frequent, but some specific symptoms such as orthostatic hypotension, hallucinations, and dementia may be present [105,106,107,108,109].

### 3.3. Omi/HtrA2 (PARK 13)

*Omi/HtrA2* (PARK13) pG399S mutation was found in a German family in 2005, followed by the discovery of other genetic variants [110,111]. The age of onset of Omi/Htra2-related PD is between 40 and 70 years [110]. Omi/HtrA2 is a serine protease with an N-terminal sequence targeting mitochondria, which can be released into the cytosol under oxidative stress condition and during apoptosis, where it suppresses the activity of some anti-apoptotic proteins [136,137].

The localization and expression of Omi/HtrA2 are tightly controlled and, therefore, mutations leading to a gain or loss of function could cause detrimental effects on several cellular processes. Indeed, mice overexpressing Omi/HtrA2 gene, Omi/HtrA2 KO mice, or p.G399S Omi/HtrA2 mutants have shown mitochondrial damage and neurodegeneration [112]. Omi/HtrA2 cooperates with parkin and PINK1 in maintaining efficient mitochondrial function and turnover [136]. Normally, Omi/HtrA2 is phosphorylated by PINK1 to enhance its protease activity, while Omi/HtrA2-overexpressing cells induce an increase in PINK1 levels, especially in the mitochondrial membrane fraction [138]. In vitro, the G399S Omi/HtrA2 mutant exhibits altered phosphorylation (at serine 400) and protease activity of Omi/HtrA2 [139], with detrimental consequences in neuronal viability. This dual localization and function—both inside the mitochondria and in the cytosol—highlights the unique pro-apoptotic profile of Omi/HtrA2, distinguishing it from other PD-related genes that primarily regulate mitochondrial dynamics or energy metabolism.

Clinical symptoms of Omi/Htra2-related PD are typical of iPD, including bradykinesia, muscular rigidity, and tremors, which are levodopa-responsive [110].

### 3.4. Vacuolar Protein Sorting 35 (VPS35) (PARK17)

*VSP35* (PARK17) gene was discovered as the third mutated gene associated with autosomal-dominant PD after SNCA and LRRK2 [140]. After the first mutation reported for *VSP35* (pD620N), several variants have been discovered, characterized by different phenotypes [113]. The age of onset of genetic PD caused by VPS35 mutations is around 50 years [116,117,141].

VSP35 is part of the retromer complex together with VPS26 and VPS29, which is critical for the retrograde transport of cargoes from the endosome to the Golgi and to the plasma membrane [142]. VPS35 was identified by Paravicini and colleagues as a gene responsible for lysosome-like vacuole assembly, and vacuolar protein sorting in Saccharomyces cerevisiae [143]. In humans, VPS35 encodes for the protein hVPS35, which is ubiquitously expressed [144].

*VSP35* pD620N mutations in neurons coming from murine substantia nigra and in patient derived-fibroblasts induced excessive mitochondrial turnover toward fission and fragmentation [115]. VPS35 mutants show increased removal of dynamin-like protein (DLP1) from mitochondria to lysosomes for degradation, altering the fusion/fission equilibrium, which is critical for mitochondrial shape and numbers. Furthermore, fibroblasts carrying VPS35 mutations showed an impairment of complex I and II activity and bioenergetics deficits [114]. This indirect control of mitochondrial dynamics through vesicular trafficking and DLP1 turnover distinguishes VPS35 from other PD genes acting more directly on mitochondrial membranes or mitophagy pathways.

Intriguingly, dopaminergic neurons derived from specific Vsp35^−/−^ mouse showed reduced viability and α-synuclein accumulation as iPD [142].

PARK17-related genetic PD is clinically similar to iPD, characterized especially by resting tremor, rigidity, bradykinesia, postural reflexes alterations, dyskinesia, autonomic symptoms, and neuropsychiatric manifestations [116,117,141].

### 3.5. Coiled-Helix-Coiled-Helix Domain Containing 2 (CHCHD2) (PARK22)

Missense mutations in *CHCHD2* (PARK22) correlate with late-onset autosomal dominant PD [118]. Indeed, a mutation in the *CHCHD2* gene (182C>T, Thr61Ile) was firstly identified in a large Japanese family with dominant PD, and then the same mutation was confirmed in other families [121,145]. The age of onset can vary among patients with PD carrying *CHCHD2* mutation, but it tends to be around 50–60 years [98].

CHCHD2 is a protein localized in the mitochondrial intermembrane space through the mitochondrial targeting sequence MTS and the cysteine-x9-cystein motifs, and it is involved in oxidative phosphorylation, mitochondria-induced apoptosis, neuronal migration, and synaptic plasticity [146]. Mutations of CHCHD2 seem to influence complex I and complex IV activity, mitochondrial biogenesis, stability, and morphology [119]. In the Drosophila model, its deletion impairs mitochondrial metabolism, causing oxidative stress, dopaminergic degeneration, and motor deficits. Intriguingly, CHCHD2 interacts with several other proteins, such as PINK1 known for its relevance with parkin in the mitophagic process [120]. This integrative role in oxidative phosphorylation, apoptosis, and neuronal development highlights CHCHD2 as a multifunctional modulator, in contrast to other PD-related genes with more specific roles in mitochondrial quality control.

From a clinical point of view, CHCHD2-related PD patients are diverse in terms of follow-up and symptoms, where some of them are characterized by an early essential tremor, restless legs syndrome, depression, and mild cognitive deficits [121].

## 4. Juvenile Genetic Atypical Parkinsonian Syndromes and Mitochondrial Dysfunction

Juvenile genetic atypical Parkinsonian syndromes represent a group of rare movement disorders that manifest at very early ages, often presenting atypical features besides the classic extrapyramidal syndrome, such as cerebellar and pyramidal symptoms, cognitive decline, ophthalmoplegia and psychiatric disorders [147]. Below, we provided a list of gene mutations involved in genetic atypical Parkinsonian syndromes, investigating their impact on mitochondrial functions (Table 3).

### 4.1. ATXN3

Spinocerebellar ataxia type 3 (SCA3), also known as Machado Joseph Disease, is the most frequent dominant genetic ataxia and it is caused by CAG repeat expansion in the *ATXN3* gene which leads to an expanded polyglutamine tract in the encoded ataxin-3 protein [148]. The age of onset varies substantially, from adolescence up to middle and older age [149,150]. This mutation results in an alteration of ataxin-3 function, a deubiquitinating enzyme relevant in the process of protein quality control. One of the substrates of ataxin-3 is E3 ubiquitin-protein ligase parkin, a key protein in mitochondrial quality control that is frequently mutated in patients with inherited juvenile PD [159,160,161]. Moreover, ataxin-3 itself was shown to be localized in the mitochondria and a calpain-mediated cleavage fragment of ataxin-3 is potentially responsible for mitochondrial fragmentation in SCA3 [151]. Furthermore, ataxin-3 has been recently shown to deubiquitinate voltage-dependent anion channel 1, a member of the mitochondrial permeability transition pore and a parkin substrate [151]. This indirect influence on mitochondrial dynamics through the deubiquitination of parkin substrates and involvement in proteostasis sets ATXN3 apart from classical PD genes that directly regulate mitophagy or mitochondrial structure. SCA3 is inherited in an autosomal-dominant way and is characterized by progressive cerebellar ataxia and variable findings including pyramidal signs, a dystonic-rigid extrapyramidal syndrome, significant peripheral amyotrophy and generalized areflexia, progressive external ophthalmoplegia, action-induced facial and lingual fasciculations, and bulging eyes [162]. Neurological findings tend to evolve as the disorder progresses. Currently, no disease-modifying treatment is available, but variable responses to antiparkinsonism agents have been reported and, recently, the benefits of deep brain stimulation (DBS) for treating SCA3 have been investigated [163].

### 4.2. CLN3

The neuronal ceroid lipofuscinoses (NCL) are a clinically and genetically heterogeneous group of neurodegenerative disorders characterized by the intra-lysosomal accumulation of autofluorescent lipopigment storage material made of proteins and lipids, including the subunit-c of the ATP synthase multi-complex [164]. They were originally divided by age onset, though they occur mostly in the first decade of life [165]. To date, 13 genes have been implicated in the various subtypes of NCL, with the CLN12 (ATP13A2) and CLN3 forms being the most commonly associated with parkinsonian symptoms. Furthermore, some rare cases of PD in patients with CLN4 and CLN11 have been described [166,167], while *CLN3* gene mutations are associated with juvenile-onset PD.

CLN3 protein is implicated in autophagy, endosomal trafficking, metabolism, and response to oxidative stress [168]. Defects in CLN3-deficient cells are shown in many compartments, such as endoplasmic reticulum, trans-Golgi network, and mitochondria [152,169,170]. In a knock-in model of neuron-like cells derived by mouse cerebellum with CLN3 mutation, ATP levels were decreased, oxidative stress was increased, and mitochondria appeared dysfunctional and elongated, affecting neuronal survival [152]. This multi-compartmental dysfunction and lysosomal origin distinguish CLN3 from classical PD genes, highlighting its involvement in atypical juvenile parkinsonism rather than in primary mitochondrial regulation.

Clinically, CLN3 is characterized by the early onset of progressive vision loss in previously healthy children followed by personality changes, behavioral problems, and slow learning. Seizures commonly appear within 2–4 years after disease onset. Progressive loss of motor functions (movement and speech) starts with clumsiness, stumbling, and Parkinson-like symptoms [153]. As far as treatments are concerned, new therapeutic approaches have been explored, and gene therapies are currently being studied [171,172].

### 4.3. GLB1

GM1 gangliosidosis is a genetic autosomal recessive lysosomal storage disorder causing relevant dysfunction in the CNS and it is caused by deficiency of β-galactosidase (β-gal) due to mutations in the GLB1 gene. It is divided into three different subtypes depending on the age onset, such as infantile, late infantile/juvenile, and adult form [154]. The infantile form is the most severe, with serious risk of death in the very early years.

*GLB1* gene codes for the lysosomal hydrolase β-gal and its mutations prevent cleavage of the terminal β-1,4-linked galactose residue from GM1 gangliosides, causing the accumulation in the lysosomes of glycolipids or other glycoconjugates that cause toxicity at different levels [154]. As shown in animal models, neurons are the primary target of these alterations, but astrocytes appear to have dysfunctional morphology as well [173]. Among the different mechanisms involved in Glb1^−/−^ mouse model of gangliosidosis, ER-stress-mediated apoptosis, dysregulation of calcium levels, enhanced autophagy, and mitochondrial dysfunction should be mentioned. In fact, in Glb1^−/−^ mice, mitochondria were smaller, fragmented or circular, with decreased membrane potential and cytochrome c oxidase activity [154]. Furthermore, cultured neurons and astrocytes were more sensitive to oxidative stress and they showed altered detoxification processes such as autophagy and mitophagy. This lysosomal origin and indirect influence on mitochondrial quality via storage-related stress pathways differentiate GLB1 from primary PD genes, highlighting its contribution as a risk modifier more than a direct effector of mitochondrial dysfunction. From a clinical point of view, subtle Parkinsonian-like symptoms might include dystonia and speech difficulty in all three forms. Interestingly, in a wide gene expression analysis in the putamen of patients with PD, the expression of GLB1 was increased, and *GLB1* mutations are considered a high genetic risk factor for iPD [174,175]. Considering the lack of effective pharmacological treatments, several clinical trials are under investigation [176].

### 4.4. POLG

*POLG* (DNA polymerase subunit gamma) mutations have been linked to different inherited mitochondrial disorders, including progressive external ophthalmoplegia, Alpers–Huttenlocher syndrome characterized by encephalopathy with intractable epilepsy and myoclonic epilepsy myopathy sensory ataxia [177,178]. POLG-related disorders consist of different phenotypes starting with early onset up to late adulthood [157]. Parkinsonism can be associated with both dominant and recessive *POLG* mutations [155]. In a study involving adult patients with mitochondrial movement disorders, 12% had parkinsonism related to *POLG* mutations, whereas POLG-related parkinsonism generally has an early onset between the third and fourth decade of life [156]. Interestingly, rare polymorphic variants of *POLG* have been suggested to be a risk factor for iPD [179,180].

POLG is the only known mammalian polymerase present in mitochondria [181], and it exerts exonuclease function, which assures the fidelity of mitochondrial DNA (mtDNA) replication and 5′ deoxyribose phosphate lyase activity [158]. The latter is instrumental for the base excision repair process necessary to correct oxidative damage to mtDNA [181,182]. This unique role in mitochondrial genome maintenance distinguishes POLG from other PD-associated genes involved in mitochondrial dynamics, highlighting a primary contribution to parkinsonism via mtDNA instability rather than altered mitophagy or respiration. Thus, POLG plays a key role in the maintenance of mtDNA. To our knowledge, *POLG* mutations are mostly used for diagnostic purposes and are currently not a therapeutic target; however, patients with POLG1 parkinsonism seem to respond to levodopa treatment for a sustained period of time [157,183].

Similarly to POLG-inherited mitochondrial disorders, another mutation related to mtDNA involves the *TWNK* gene which encodes for the mitochondrial twinkle helicase, which is fundamental in the process of mtDNA replication [184]. *TWNK* variants have been associated with different pathological phenotypes, including autosomal dominant progressive external ophthalmoplegia (adPEO), whereas some patients also presented late onset parkinsonism [185,186].

### 4.5. Hereditary Spastic Paraplegia (HSP)

Hereditary spastic paraplegia (HSP) is characterized by the progressive degeneration of corticospinal tracts. To date, nine loci are known to be involved and are classified as spastic paraplegia genes (*SPG1–SPG79*) [187]. SPG7 encodes paraplegin, a mitochondrial inner-membrane metalloprotease involved in OPA1 cleavage, a protein that regulates mitochondrial fission/fusion processes and mitochondrial cristae structure [188,189,190]. Paraplegin regulates mitochondrial ribosomes and, thus, protein synthesis [191]. It also seems to be a regulator of the mitochondrial permeability transition pore [192,193]. Paraplegin is also involved in axonal development [194]. SPG7 forms heterooligomeric protease complexes with the homologous ATPase AFG3L2 [188]. This complex is involved in axonal development and consequences of an impaired complex include mitochondrial dysfunction [188,194]. Mutations in *SPG7* gene result in multiple mtDNA deletions, and manifest phenotypically as mitochondrial disorders characterized by spasticity, ataxia, dysarthria, dysphagia, cognitive impairment, neuropathy, ophthalmoplegia, muscle wasting, sphincter dysfunction, and parkinsonism [195,196]. Particularly, data from 241 European patients with *SPG7* alterations found out that the predominance of pyramidal signs and symptoms is associated with the presence of homozygous loss of function variants rather than missense mutations, suggesting that the loss of paraplegin function drives spasticity [196]. This unique link between mitochondrial proteostasis, cristae architecture, and axonal development differentiates SPG7 from PD genes that primarily regulate mitophagy or respiratory function, and places it within a broader neurodegenerative spectrum associated with atypical parkinsonism.

In 2018, levodopa-responsive parkinsonism has been described in two HSP caused by *SPG7* alterations (SPG7-HSP) patients [197,198]. The following year, another independent study analyzed a cohort of SPG7-HSP patients and found out that 21% (*n* = 7) of them showed parkinsonian signs such as bradykinesia, tremor, or rigidity. Three patients were treated with L-dopa and all of them responded to this treatment [199].

A collaborative international study found that early-onset patients with PD were heterozygous carriers of the new variant p.Ala510Val. The frequency of this variant was significantly higher among patients in wide genetic databases, suggesting SPG7 as a novel candidate gene for early-onset PD [200]. Nevertheless, the putative pathogenic role of SPG7 variants in iPD is still to be fully elucidated [199].

## 5. Environmental Toxins, PD, and Mitochondrial Dysfunction

While genetic PD represents approximately 10% of all PD cases, the remaining 90% are sporadic, caused by unknown factors. Among these factors, environmental toxins have received much attention, suggesting a credible interplay between environmental and genetic factors in the etiology of the different forms of PD [14,201,202]. Several epidemiological studies have proposed a correlation between living in rural areas with pesticides exposure and PD incidence, supporting a role for toxin exposure in the etiology of the disease [203,204]; however, this correlation has not always been confirmed [205].

Historically, in the early 80s, the first toxin found to induce PD was 1-methyl-4-phenyl-1,2,3,6-tetrahydropyridine (MPTP), a contaminant of illicit synthetic opioid that was synthetized accidentally by a drug abuser [206]. The hypothesis that environmental toxins might contribute to PD was strengthened by the observation that the herbicide paraquat, structurally similar to MPTP, and the natural pesticide rotenone can be neurotoxic and produce extrapyramidal symptoms in human and animal models [14]. MPTP is a highly lipophilic molecule that easily crosses the blood–brain barrier, and then it is transformed to its toxic metabolite MPP+, which is selectively uptaken by dopamine transporters (DATs) into dopaminergic neurons; then, it is concentrated into synaptic vesicles by vesicular monoamine transporter type 2 (VMAT2) [207]. MPTP induces greater dopaminergic neurodegeneration in the substantia nigra than in the ventral tegmental area, firstly in the striatal dopaminergic nerve terminals and secondly in the cell body, due to higher DAT, VMAT2, and mitochondrial density in the synapse compared to the soma [208]. MPTP toxicity is generally applied to mouse and primate PD models, reproducing in part the human physiopathology, although elements such as Lewy body inclusion and α-synuclein aggregates have not been replicated consistently [209]. MPP+ acts by inhibiting complex I of the mitochondrial respiration chain, thus leading to reduced ATP production, ROS generation, and dissipation of the MMP, which trigger mitochondrial dysfunction and fragmentation, generating a vicious cycle that leads to apoptosis [210,211].

Similarly to MPTP, rotenone, now banned in numerous countries due to its neurotoxicity in humans, is a potent mitochondrial complex I inhibitor, and it is widely used in rodent models of PD [212,213]. In vitro and in vivo exposure to rotenone increases ROS production, mitochondrial dysfunction, activation of microglia, and apoptosis, together with ATP reduction and electron leakage [9,214,215]. The model of rotenone-induced toxicity reproduces Lewy body inclusion and α-synuclein aggregates more reliably compared to MPTP model, probably because of its strong potency for inhibiting mitochondrial activity and/or for involving other mechanisms such as α-synuclein phosphorylation, inactivation of the Akt/mTOR signaling, and increasing mtDNA mutations [213,216].

While MPP+ and rotenone inhibit complex I during forward electron transfer, producing ROS, other compounds, such as metformin, inhibit it during reverse electron transfer without causing oxidative stress [217]. In light of this, metformin is increasingly studied for its potential neuroprotective effects in MPTP-caused PD models [218].

Mitochondrial dysfunction and ROS overproduction are extremely deleterious for dopaminergic neurons, which are particularly vulnerable to oxidative stress due to their high metabolic activity and wide arborization at their terminals [219,220]. Consistently, prolonged oxidative stress results in the loss of dopaminergic neurons, a crucial event for PD progression [16,17]. According to some intriguing hypothesis, dopaminergic neurons might also be damaged by oxidative stress coming from endogenous substances, such as dopamine itself and its metabolites. In fact, in the cytosol and in the synaptic cleft of neurons, dopamine metabolism produces several oxidants due to enzymatic and non-enzymatic reactions, such as H_2_O_2_, •O_2−_ and hydroxyl (•OH) and dopamine-semiquinone radicals [221,222]. Additionally, the presence of high amount of iron in the substantia nigra of PD can contribute to ROS and α-synuclein aggregate production [216]. Indeed, mitochondria are important for iron exchange with the cytosol to integrate iron–sulfur clusters, part of complex I and II, and its accumulation can have a negative impact on mitochondrial dynamics [223,224].

Similarly to dopamine, the chronic use of the dopamine precursor L-DOPA can increase ROS production and oxidative stress, and for this reason clinicians might consider using this compound later during the progression of the disease, when the symptoms become more severe and the treatment with dopamine agonists is not satisfactory anymore [225].

If the toxicity of dopamine and L-DOPA is still questionable, the dopamine analog 6-OHDA is a well-known neurotoxin used as a PD model in rodents, and accumulates in catecholaminergic neurons through the dopaminergic and noradrenergic transporters [226,227]. In vitro models have shown how 6-OHDA is capable of increasing ROS production, membrane permeability, and mitochondrial dysfunction, underlying the close connection between oxidative stress and mitochondrial activity in a bidirectional, vicious cycle that can generate neuronal death (Table 4).

## 6. Converging Mechanisms in Parkinson’s Disease: The Mitochondrial Link Between Genetic and Non-Genetic Cases

Mitochondrial dysfunction represents a well-established key element in the pathogenesis of genetic and non-genetic PD, and it is involved in iPD as well. Initially originating from the observation that mitochondrial toxins cause PD by inhibiting complex I function, evidence accumulated over the past decades has clearly demonstrated that several monogenic mutations involved in early- and late-onset PD directly impair mitochondrial functions. Notably, some of these genetic mutations alter the endo-lysosomal-proteasome system as well, creating a vicious cycle of cellular impairment with the accumulation of aberrant proteins (Figure 2). If on one side impaired lysosomal degradation causes an accumulation of dysfunctional mitochondria in PD [3], on the other side mitochondrial deficits are associated with ROS overproduction and insufficient capability to supply many cellular processes that require high levels of energy, including autophagy.

These monogenic mutations involve proteins that regulate the most important activities in the mitochondrial life, such as its enzymatic activity, trafficking, clearance, and turnover.

Particularly, single genes causing early-onset PD, such as PINK1, Parkin, *DJ-1*, ATP13A2, PLA2G6, and FBXO7, encode for proteins involved in mitochondrial quality control, through processes like mitophagy, turnover, maintenance of morphology, and finally stress responses.

Additionally, genes causing late-onset PD such as SNCA, LRRK2, and VPS35 affect mitochondrial function through alterations in mitochondrial dynamics, bioenergetics, and trafficking. It is worth mentioning that these mutated proteins have a detrimental effect on other important cellular functions related to the autophagy process to target dysfunctional cargoes to the lysosomes through the UPS.

Regarding the juvenile atypical parkinsonian syndromes, mutated genes such as ATXN3, CLN3, GLB1, and POLG cause mitochondrial impairment with a broad spectrum of systemic and neurodegenerative features. These genes are involved in mitochondrial homeostasis, lipid metabolism, and lysosomal function, and their dysfunction results in profound mitochondrial fragmentation, impaired mitophagy, and oxidative damage, usually from a very early age [151,153,154,156].

As a confirmation of the relevance of mitochondrial integrity in PD, environmental toxins such as MPTP, rotenone, and paraquat also mimic PD-related genetic damage by acting on complex I of the respiratory chain and generating excessive ROS, pointing out mitochondria and oxidative stress as a common pathogenic link. Rotenone and 6-OHDA models further replicate key pathological features such as α-synuclein aggregation, providing mechanistic overlap with both early- and late-onset PD.

**Figure 3:**
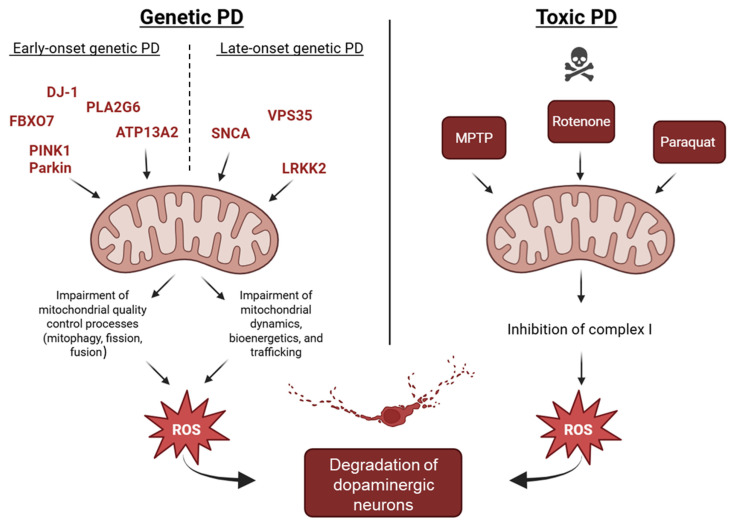
Mitochondrial processes in genetic, non-genetic, and toxic forms of Parkinson’s disease (PD). The gene mutations associated with early- and late-onset PD significantly impair mitochondrial quality control mechanisms, including mitophagy, mitochondrial dynamics, and bioenergetics (left panel). Environmental toxins such as MPTP, rotenone, and paraquat inhibit mitochondrial complex I activity (right panel). In genetic- as well as toxin-induced PD, the resulting accumulation of reactive oxygen species (ROS) leads to oxidative stress and ultimately contributes to dopaminergic neuron degeneration.

Importantly, dopaminergic neurons of the substantia nigra, due to their extended arborization and high energy demand, are very sensitive to ROS production and oxidative stress, which is strongly connected with mitochondrial dysfunction [231]. In both genetic and non-genetic PD forms, the vulnerability of dopaminergic neurons in the substantia nigra is amplified by their high energy demand and consequent oxidative stress (Figure 3). Thus, oxidative stress, resulting from or amplified by mitochondrial deficits, is a crucial driver of dopaminergic neurodegeneration.

Collectively, these observations point out to mitochondrial impairment as a unifying feature across both genetic and toxic forms of PD. Whether initiated by gene mutations or environmental insults, mitochondrial dysfunction drives neuronal degeneration through converging pathways of oxidative stress, defective bioenergetics, and impaired organelle quality control.

## 7. Mitochondria-Targeted Therapeutic Approaches in PD

Mitochondria-targeted therapeutic approaches in PD focus on restoring mitochondrial function and reducing oxidative stress, both of which contribute to dopaminergic neuronal degeneration in the substantia nigra. Strategies include the use of antioxidants specifically delivered to mitochondria, agents that enhance mitochondrial biogenesis and dynamics, and molecules that stabilize mitochondrial membranes or modulate the mitochondrial permeability transition pore. Additionally, some approaches aim to improve mitophagy, the process by which damaged mitochondria are removed, thereby preventing the accumulation of dysfunctional organelles that contribute to neuronal death. Table 5 summarize the main mitochondria-targeted strategies in PD.

Among the most extensively studied strategies are compounds that enhance electron transport chain (ETC) activity or bypass its dysfunction. Coenzyme Q10 (ubiquinone) and its synthetic analog idebenone, similarly, have been shown to improve mitochondrial electron flow and reduce oxidative damage in preclinical PD models [233,234]. However, despite their biochemical promise, clinical trials have produced inconsistent results, possibly due to issues related to bioavailability and limited blood–brain barrier penetration [235]. Methylene blue has emerged as a novel redox-active compound capable of shuttling electrons in the ETC independently of complex I, providing neuroprotection and improving both motor and non-motor outcomes in toxin-induced PD models [236]. Likewise, creatine has demonstrated beneficial effects in maintaining ATP homeostasis, though its efficacy in human trials remains limited [237,238].

Beyond ETC-targeting agents, several naturally occurring phytochemicals exhibit promising mitochondrial effects. Resveratrol, curcumin, and quercetin—all polyphenolic antioxidants—have been shown to promote mitochondrial biogenesis, reduce oxidative stress, and modulate mitochondrial dynamics. Resveratrol, in particular, activates SIRT1 and PGC-1α, key regulators of mitochondrial biogenesis, and has demonstrated synergistic effects when combined with L-DOPA therapy [239]. However, challenges such as poor solubility and rapid metabolism have led to the exploration of nanoparticle formulations to enhance their therapeutic potential [240].

The modulation of mitochondrial homeostasis by bile acids has also garnered interest. Ursodeoxycholic acid (UDCA), a hydrophilic bile acid, has shown the ability to restore mitochondrial membrane potential, enhance ATP production, and reduce oxidative damage in both in vitro and in vivo PD models [241,242]. Similarly, interventions targeting iron accumulation—a known contributor to mitochondrial stress in PD—such as deferiprone and deferoxamine, offer neuroprotection by restoring metal homeostasis and reducing iron-mediated ROS production [243].

Peptides and endogenous regulators also play a crucial role in mitochondrial modulation. Among these, antidiabetic Glucagon-like peptide-1 (GLP-1) receptor agonists, such as exendin-4, have been shown to enhance mitochondrial biogenesis via PKA and MAPK/AKT signaling pathways. These agents not only improve mitochondrial efficiency but also inhibit apoptosis, though clinical trials have yet to demonstrate significant functional improvement in PD patients. Sirtuins, particularly SIRT1 and SIRT3, are NAD^+^-dependent deacetylases involved in mitochondrial stress responses and metabolic regulation [244]. While their activation shows promise in preclinical studies, therapeutic translation remains limited [245].

Further mitochondrial support is provided by targeting intracellular signaling pathways associated with bioenergetics and antioxidant responses. Modulators of the AMPK-SIRT1-PGC-1α (adenosine monophosphate-activated protein kinase/sirtuin1/proliferator-activated receptor gamma coactivator-1 alpha) axis, such as ferulic acid, RNS60, and the mitochondria-targeted antioxidant MitoQ, have demonstrated efficacy in enhancing mitochondrial biogenesis and fusion, thereby improving neuronal survival in PD models [246]. MitoQ, in particular, stabilizes mitochondrial membranes and promotes fusion via mitofusin-2 (Mfn2) regulation [247]. Additionally, compounds that activate the Nrf2/ARE pathway, such as TPNA10168, have been found to induce the expression of antioxidant enzymes like HO-1 and NQO1, effectively mitigating oxidative damage [248].

Apoptosis, driven by mitochondrial dysfunction, is another critical therapeutic target. Compound A, by inhibiting succinate dehydrogenase (complex II), reduces cytochrome c release and prevents caspase-dependent neuronal death [249]. Likewise, the small molecule Mdivi-1 has gained attention for its ability to inhibit dynamin-related protein 1 (Drp1), a key mediator of mitochondrial fission. By preventing excessive mitochondrial fragmentation, Mdivi-1 restores mitochondrial morphology and reduces ROS accumulation in PD models [250].

Mitophagy—the selective clearance of damaged mitochondria—is frequently impaired in PD due to mutations in genes such as PINK1 and Parkin. Pharmacological enhancement of mitophagy represents a promising strategy to eliminate dysfunctional organelles and restore cellular homeostasis. Compounds like IU1, which inhibits the proteasomal regulator USP14, enhance mitochondrial quality control and protect against dopaminergic degeneration. However, many traditional mitophagy inducers, such as FCCP or oligomycin–antimycin combinations, remain unsuitable for clinical use due to their cytotoxicity [251].

Emerging experimental approaches include novel molecular targets such as P13, a mitochondrial protein that promotes apoptosis in neurotoxin-challenged neurons. Downregulation of P13 has been shown to attenuate dopaminergic cell death and restore mitochondrial function [252]. Interestingly, proteins derived from the Borna disease virus (BDV), particularly protein X, have been reported to protect mitochondrial integrity and prevent apoptosis, although this area of research remains largely exploratory [253].

Finally, a highly innovative approach involves mitochondrial transplantation. The transfer of viable, allogeneic mitochondria into affected tissues or the bloodstream has been shown to restore mitochondrial respiration, reduce oxidative stress, and improve dopaminergic neuron survival in preclinical models of PD [254]. While still in the early stages, this technique offers a promising platform for future translational therapies [255,256].

In conclusion, mitochondrial boosts represent a multifaceted and evolving field of neuroprotective strategies in Parkinson’s disease. While most interventions remain in the preclinical or experimental phase, their mechanistic diversity and potential to restore mitochondrial function provide compelling avenues for the development of disease-modifying treatments.

## 8. Conclusions and Future Perspectives

Mitochondrial dysfunction has been clearly established as a unifying pathogenic mechanism of genetic and non-genetic PD, and it is associated with ROS overproduction, apoptosis, and an insufficient capability to supply many cellular processes that require high energy demands, including the endo-lysosomal-proteasome system.

Notably, several monogenic mutations in early- and late-onset PD encode for proteins that are either expressed in the mitochondria or regulate the most important organelle properties, such as the ETC, trafficking, morphology, integrity, and, lastly, mitophagy. In addition, other atypical juvenile genetic forms of PD alter mitochondrial activities and cause mitochondrial fragmentation, impaired mitophagy, and oxidative damage, usually from a very early age [151,153,154,156]. For instance, dopaminergic neurons, due to their extended arborization and high energy demand, are very sensitive to ROS production and oxidative stress, which is strongly connected to mitochondrial dysfunction [257].

Despite the recognition of these processes, currently available therapies are not yet able to modify the pathological course of PD. For this reason, mitochondria dysfunctions, whether as a cause or consequence of PD, represent potential targets for therapeutic strategies aimed at slowing disease progression and uncovering players involved in these processes will be crucial for developing more personalized therapeutic strategies. Among the novel treatments aimed at improving mitochondrial function are the use of antioxidants specifically delivered to mitochondria, agents that enhance mitochondrial biogenesis and dynamics, and molecules that increase mitophagy, the process by which damaged mitochondria are removed. These therapies offer promising avenues to slow or halt disease progression by targeting a core pathological mechanism of PD.

## Figures and Tables

**Table 1:** Early-onset genetic PD.

Gene	Protein and Mutation Type	Shared MT Alterations	Gene-Specific MT Alterations	Age of Onset (Motor Symptoms)	Clinical Consequences
**Parkin (PARK2)** [27,28,29,30,31]	E3 ubiquitin ligase AR (more than 130 mutations)	↓ MMP ↓ Complex I activity ↓ ATP ↑ ROS	↓ complex III activity ↑ complex II activity (compensatory) UPS dysfunction (↓ protein degradation) PINK1-dependent mitophagy MDV formation Loss of MitAP inhibition (↑ MitAP and immune activation)	<40 years	Slow progression, dystonia; infrequent olfactory dysfunction and cognitive impairment
**PINK1 (PARK6)** [27,30,31,32,33,34,35,36]	MT serine-threonine kinase AR (111 point, frameshift, and truncating mutations)	↓ OXPHOS; ↓ MT respiration; Altered MT morphology ↓ MT fission → MT swelling/enlargement	Outer membrane accumulation Parkin/ubiquitin phosphorylation MDV formation Loss of MitAP inhibition (↑ MitAP and immune activation)	Mid-30s	Slow progression, dystonia, non -motor symptoms, occasionally psychiatric disorders
***DJ-1* (PARK7)** [37,38,39,40]	Peptidase C56 family AR (homozygous deletions or point mutations)	Altered MT morphology ↓ respiratory chain complex activity ↓ MMP ↑ ROS	↓ UCP4/UCP5 activity ↓ Bcl-xL interaction → ↑ apoptosis	<50 years	Slow progression, early onset of motor symptoms, amyotrophy, cognitive impairment, acute behavioral disturbances
**ATP13A2 (PARK9)** [41,42,43,44]	Neuronal lysosomal type 5 P-type ATPase AR (loss-of-function mutations)	↓ ATP ↑ MT oxidative stress MT fragmentation	Impaired lysosomal polyamine transport ↓ TOM20 expression ↓ Autophagosome–lysosome fusion Lysosome–MT crosstalk dysfunction	<20 years	Dementia, pyramidal degeneration, spasticity, supranuclear gaze palsy
**PLA2G6 (PARK14)** [3,45,46,47]	Ca^2+^-independent phospholipase A2β AR (more than 18 variants)	↓ ATP ↑ ROS ↓ MMP ↓ Complex I activity MT membrane abnormalities	MT lipid peroxidation ↓ Cytochrome c oxidase ↓ SOCE signaling → ↓ intracellular Ca^2+^ → autophagic impairment ↓ Parkin protein levels Phospholipid membrane remodeling dysfunction	Adolescence-early 20s	Dystonia, gait impairment, speech difficulties, spasticity, myoclonus, neuropsychiatric and cognitive disorders
**FBXO7 (PARK15)** [48,49,50,51,52]	F-box proteins (FBPs) adaptor protein member AR (3 point mutations, homozygous truncating FBXO7 mutation, compound heterozygous mutations)	↓ MMP ↓ ATP levels ↓ Complex I activity ↑ ROS	↓ NAD^+^ levels ↑ PARP activation → NAD^+^/ATP depletion	Childhood	Tremor, rigidity, bradykinesia, pyramidal signs
**VPS13C (PARK23)** [53,54]	Vacuolar sorting proteins 13 family AR (truncating mutations)	↓ MMP MT fragmentation ↑ ROS	↑ Parkin transcription ↑ PINK1/Parkin-dependent mitophagy Disrupted MT–peroxisome trafficking Impaired ER–MT–lysosome crosstalk	Early 20s	Lewy-body inclusions, cognitive decline, axial symptoms, dysautonomia

Abbreviations: AR: autosomal recessive; MT: mitochondria/mitochondrial; MMP: mitochondrial membrane potential; ROS: reactive oxygen species; OXPHOS: oxidative phosphorylation; ATP: adenosine triphosphate; UCP: uncoupling protein; SOCE: store-operated calcium entry; SCF: Skp1–Cullin–F-box complex; PARP: poly(ADP-ribose) polymerase; MDV: mitochondrial-derived vesicle; MitAP: mitochondrial antigen presentation; ↓: reduction; ↑: increase.

**Table 2:** Late-onset genetic PD.

Gene	Protein and Mutation Type	Shared MT Alterations	Gene-Specific MT Alterations	Age of Onset (Motor Symptoms)	Clinical Consequences
**SNCA** (**PARK1/** **PARK4**) [15,94,95,96,97,98]	α-synuclein AD (p.A53T, p.A30P, p.E46K, p.G51D, p.A53E, duplications, triplications)	↓ MMP ↑ ROS MT fragmentation	Complex I inhibition (via N-terminal α-syn domain) ↓ MT protein import (via TOM20 binding) ↑ Phospho-α-syn → energy deficit Altered mitophagy (p.A53T mutant)	20–85 years	Resting tremor, bradikinesia, rigidity, dysphagia, dysarthria, cognitive deficits
**LRRK2** **(PARK8)** [99,100,101,102,103,104,105,106,107,108,109]	Leucine-rich repeat kinase 2 AD (G2019S)	↓ ATP ↑ ROS MT fragmentation	↓ Complex III and IV activity Delayed mitophagy (↓ clearance of damaged MT) Arrested fission (via Drp1 interaction)	30–80 years	Bradykinesia, rigidity resting tremor, gait abnormalities, postural instability, orthostatic hypotension, hallucinations, dementia, less frequent RBD and anosmia
**Omi/HtrA2 (PARK 13)** [110,111,112]	Serine protease (p.G399S and other genetic variants)	MT damage	Altered protease activity (p.G399S mutant) → ↓ MT viability Impaired phosphorylation → ↓ quality control/↑ neuronal stress Disrupted cooperation with PINK1/parkin → ↓ MT turnover	40–70 years	Bradykinesia, muscular rigidity, tremor
**VPS35** **(PARK17)** [113,114,115,116,117]	hVPS35 AD (p.D620N and other mutations)	↓ Complex I and II activity ↓ Bioenergetic efficiency	↑ MT fission via DLP1 degradation → fragmentation Vesicle trafficking defects (retromer dysfunction) → indirect MT stress	Around 50 years	Resting tremor, rigidity, bradykinesia, postural reflexes alterations
**CHCHD2 (PARK22)** [118,119,120,121]	CHCHD2 AD (missense mutations)	↓ Complex I activity ↑ oxidative stress ↓ MT stability/morphology	↓ Complex IV activity ↓ MT biogenesis ↑ MT metabolic dysfunction Dysregulation of MT apoptosis pathways	Mid-50s	Early essential tremor, restless legs syndrome, depression, mild cognitive deficits

Abbreviations: AD: autosomal dominant; MT: mitochondria/mitochondrial; ROS: reactive oxygen species; MMP: mitochondrial membrane potential; TOM20: translocase of the outer mitochondrial membrane 20; α-syn: alpha-synuclein; Drp1: dynamin-related protein 1; RBD: REM sleep behavior disorder; PINK1: PTEN-induced kinase 1; ↓: reduction; ↑: increase.

**Table 3:** Juvenile genetic PD and parkinsonisms.

Gene	Protein and Mutation Type	Shared MT Alterations	Gene-Specific MT Alterations	Age of Onset (Motor Symptoms)	Clinical Consequences
**ATXN3** [148,149,150,151]	Ataxin-3 protein AD (CAG repeat expansion)	↑ ROS MT fragmentation	Impaired parkin-mediated mitophagy (via deubiquitination) ↑ Mitochondrial permeability (VDAC1 dysfunction)	Adolescence-middle age	Progressive cerebellar ataxia, pyramidal signs, dystonic-rigid extrapyramidal syndrome, peripheral amyotrophy, generalized areflexia, external ophthalmoplegia, action-induced facial and lingual fasciculations, bulging eyes
**CLN3** [152,153]	CLN3 protein AR	↓ ATP levels ↑ oxidative stress	MT elongation Impaired MT function secondary to lysosomal and ER-Golgi trafficking defects	Childhood (4–7 years)	Early-onset progressive vision loss, personality changes, behavioral problems, slow learning, seizures, progressive motor function loss
**GLB1** [154]	β-galactosidase (β-gal) AR (cleavage of the terminal β-1,4-linked galactose residue from GM1 gangliosides)	↑ Oxidative stress ↓ MMP Altered autophagy/ mitophagy	Abnormal MT morphology ↓ Cytochrome c oxidase activity MT dysfunction secondary to lysosomal storage pathology	Childhood-adolescence	Dystonia/hypotonia, speech difficulty, hepatosplenomegaly, developmental regression, seizures, visual impairment
**POLG** [155,156,157,158]	DNA Polymerase subunit gamma	↑ Oxidative stress ↓ ATP production ↓ Respiratory chain activity (OXPHOS impairment)	mtDNA deletions and depletion ↓ mtDNA replication fidelity ↓ base excision repair capacity mtDNA instability as primary pathogenic mechanism	Early childhood to third–fourth decade	Various clinical features depending on the specific syndrome

Abbreviations: AD: autosomal dominant; AR: autosomal recessive; MT: mitochondria/mitochondrial; ROS: reactive oxygen species; MMP: mitochondrial membrane potential; ATP: adenosine triphosphate; OXPHOS: oxidative phosphorylation; VDAC1: voltage-dependent anion channel 1; ER: endoplasmic reticulum; UPS: ubiquitin–proteasome system; MDV: mitochondria-derived vesicles; mtDNA: mitochondrial DNA; SCF: Skp—Cullin—F-box-containing complex; Bcl-xL: B-cell lymphoma—extra-large; SOCE: store-operated calcium entry; UCP: uncoupling protein; TOM20: translocase of outer mitochondrial membrane 20; SN neurons: substantia nigra neurons; NAD^+^: nicotinamide adenine dinucleotide; PARP: poly(ADP-ribose) polymerase; ↓: reduction; ↑: increase.

**Table 4:** Toxic PD.

Toxic Agent	Toxin Type	First Identification	Mitochondrial Alterations
**Rotenone** [208,214,228,229,230]	Crystalline isoflavone, used as pesticide, insecticide, and piscicide	1990s epidemiological studies in humans; first in vivo PD model in rats in 2000	Systemic MT complex I inhibitor; ↑ ROS production; ↓ ATP production; Electron leakage; MT dysfunction; Microglia activation; Apoptosis.
**MPTP** [206,207,210,231]	Tetrahydropyridine, precursor of MPP+	Late 1970s–early 1980s toxicity found in humans (after contaminated intravenous drug use); first animal model in 1984 (squirrel monkey)	MT complex I inhibitor; ↑ ROS production; ↓ ATP production; MMP dissipation; MT dysfunction and fragmentation; Apoptosis.
**6-OHDA** [226,232]	Dopamine-derived benzenetriol	Toxicity described in 1959; first PD (akinesia) model in 1968	↑ ROS production; ↑ membrane permeability; Mitochondrial dysfunction; Apoptosis.

MPTP: 1-methyl-4-phenyl-1,2,3,6-tetrahydropyridine; MPP+: 1-methyl-4-phenylpyridinium; 6-OHDA: 6-hydroxydopamine; ROS: reactive oxygen species; ATP: adenosine triphosphate; MT: mitochondrial; MMP: mitochondrial membrane potential; ↓: reduction; ↑: increase.

**Table 5:** An overview of the main mitochondria-targeted strategies in PD.

Category	Agents	Mechanism of Action	Evidence Level
**ETC and Antioxidants**	CoQ10, Idebenone, Methylene Blue, Creatine	Bypass complex I, ETC facilitation, ROS neutralization	Preclinical and Limited Clinical
**Phytochemicals**	Resveratrol, Curcumin, Quercetin	Antioxidant activity, mitophagy, mitochondrial fusion	Preclinical
**Bile Acids**	UDCA, Taurine-UDCA	Enhances ATP, reduces ROS, stabilizes membrane potential	Preclinical
**Metal Homeostasis**	Deferiprone, Deferoxamine	Chelation of Fe, Cu; reduces oxidative stress	Preclinical
**Peptides/Proteins**	GLP-1 Agonists, SIRT1/SIRT3	Mitochondrial biogenesis, metabolic regulation	Preclinical and Mixed Clinical
**Signaling Modulators**	MitoQ, Ferulic Acid, RNS60, TPNA10168	Biogenesis via AMPK/PGC-1α, antioxidant response via Nrf2/ARE	Preclinical
**Mitochondrial Dynamics**	Mdivi-1, Compound A	Inhibits fission and apoptosis pathways	Preclinical
**Mitophagy**	IU1	Induces selective clearance of dysfunctional mitochondria	Preclinical
**Experimental Approaches**	P13 Inhibition, BDV X Protein	Apoptosis regulation, mitochondrial integrity restoration	Experimental
**Mitochondrial Transplantation**	Allogeneic Mitochondria	Mitochondrial replacement therapy	Preclinical

ETC: electron transport chain; CoQ10: coenzyme Q10; ROS: reactive oxygen species; UDCA: ursodeoxycholic acid; GLP-1: Glucagon-like peptide-1; SIRT1/SIRT3: sirtuin 1 and 3; AMPK: AMP-activated protein kinase; PGC-1α: peroxisome proliferator-activated receptor gamma coactivator 1-alpha; Nrf2/ARE: nuclear factor erythroid 2–related factor 2/antioxidant response element; IU1: inhibitor of ubiquitin-specific protease 14; BDV: Borna disease virus; ATP: adenosine triphosphate.
